# THE EFFECT OF SELF-PERCEPTIONS OF AGING ON PHYSICAL ACTIVITY: RESULTS FROM THE AGINGPLUS STUDY

**DOI:** 10.1093/geroni/igac059.1677

**Published:** 2022-12-20

**Authors:** Abigail Nehrkorn-Bailey, Han-Yun (Heidi) Tseng, Diana Rodriguez, Kaigang Li, George Rebok, David Roth, Shang-En Chung, Manfred Diehl

**Affiliations:** University of Wisconsin-Green Bay, Green Bay, Wisconsin, United States; Colorado State University, Fort Collins, Colorado, United States; Colorado State University, Fort Collins, Colorado, United States; Colorado State University, Fort Collins, Colorado, United States; Johns Hopkins Bloomberg School of Public Health, Baltimore, Maryland, United States; Johns Hopkins University, Baltimore, Maryland, United States; Johns Hopkins University, Baltimore, Maryland, United States; Colorado State University, Fort Collins, Colorado, United States

## Abstract

The AgingPLUS program targets negative self-perceptions of aging (SPA) as one mechanism to increase physical activity (PA) in adults. This study utilized a mediation model to examine the effect of AgingPLUS on subsequent PA with SPA included as a mediator. Data came from 184 participants (Mage = 59.91 years; SDage = 8.14 years) from the ongoing trial. Although the direct effect from condition to Week 8 PA was not significant, the pathway from condition to Week 4 SPA was significant. Additionally, the pathway from Week 4 SPA to Week 8 PA was marginally significant (β = .11, p = .07). However, the indirect effect was not significant. Given that (1) the AgingPLUS program resulted in significantly more positive SPA and (2) more positive SPA marginally predicted more minutes of subsequent PA, these results provide preliminary support for the efficacy of the ongoing program.

